# Regulating digital health technologies with transparency: the case for dynamic and multi-stakeholder evaluation

**DOI:** 10.1186/s12916-019-1447-x

**Published:** 2019-12-03

**Authors:** Elena Rodriguez-Villa, John Torous

**Affiliations:** Department of Psychiatry, Beth Israel Deaconess Medical Center, Harvard Medical School, 75 Fenwood Road, Boston, MA 02115 USA

**Keywords:** Digital health, mhealth, Regulation, Smartphone apps, ehealth

## Abstract

**Background:**

The prevalence of smartphones today, paired with the increasing precision and therapeutic potential of digital capabilities, offers unprecedented opportunity in the field of digital medicine. Smartphones offer novel accessibility, unique insights into physical and cognitive behavior, and diverse resources designed to aid health. Many of these digital resources, however, are developed and shared at a faster rate than they can be assessed for efficacy, safety, and security—presenting patients and clinicians with the challenge of distinguishing helpful tools from harmful ones.

**Main text:**

Leading regulators, such as the FDA in the USA and the NHS in the UK, are working to evaluate the influx of mobile health applications entering the market. Efforts to regulate, however, are challenged by the need for more transparency. They require real-world data on the actual use, effects, benefits, and harms of these digital health tools. Given rapid product cycles and frequent updates, even the most thorough evaluation is only as accurate as the data it is based on.

**Conclusions:**

In this debate piece, we propose a complementary approach to ongoing efforts via a dynamic self-certification checklist. We outline how simple self-certification, validated or challenged by app users, would enhance transparency, engage diverse stakeholders in meaningful education and learning, and incentivize the design of safe and secure medical apps.

## Background

The unmet need for psychiatric services has accelerated interest in technologies such as mobile apps to bridge the mental health gap. With worldwide ownership of smartphones already at 2.5 billion [1], the opportunity to utilize these devices to screen, assess, monitor, and even intervene in psychiatric conditions is unprecedented. The potential for this new generation of accessible, affordable, and accurate digital mental health tools has already attracted the attention of the public, large technology companies, and national healthcare regulators.

The attention on medical apps is significant, innovation so novel, and product development so fast as to overwhelm current regulatory systems. The 10,000 mental health apps available for immediate download on the iTunes and Android stores [2] offer a concrete representation of the rapid pace of development. Innovative apps for therapy, medication adherence, and mindfulness are now a few clicks away for billions of people around the world. Case reports and early efficacy studies suggest clinical benefits in research settings [3, 4]. Yet examination of the less tangible aspects of these apps, including lack of clinical evidence for many [5, 6], clinical safety concerns for some [7], and emerging privacy vulnerabilities for most [8], offer a second perspective. For example, apps that appear effective in research settings do not always appear to be equally efficacious in real-world clinical settings [9, 10]. While digital health tools may serve the unmet needs of tech savvy people well, they may not meet the needs of, and even inadvertently discriminate against, those who are not technology or smartphone literate. Ensuring digital health equity and realizing the potential of increased access and innovation with mental health apps must thus be balanced with a rapidly evolving marketplace, scientific evidence, and unknown risks [11]—presenting a novel challenge for regulation.

## Main text

### Steps to regulate

Growing pressure to inform the public around the safety and efficacy of new innovations in apps and other digital health technologies has prompted initial evaluation efforts. In 2015, the US Food and Drug Administration (FDA) released formal guidelines on its approach to regulating “Mobile Medical Apps” [12]. The guidelines function as a hierarchy. They prioritize monitoring and the approval process of mobile apps that directly control medical devices or function as these devices on their own. Mobile apps that are educational or promote “wellness” are considered less harmful. They pose an ostensibly smaller risk to public safety and health and therefore require less or no oversight. This strategy, explained with the concept of regulatory discretion, effectively narrows the scope of mobile applications the FDA oversees and approves. But in the case of mental health, regulatory discretion presents unique challenges as it excludes many or most mental health-related resources from evaluation. Thus, while many mental health-related apps make claims that appear medical or that a reasonable consumer might interpret as clinical [13], these digital tools are not subject to regulation or enforcement of privacy and confidentially protection for patients.

The UK’s National Health Service (NHS) utilizes a different approach towards offering guidance and protections around mobile health apps. The NHS Health Apps Library is a repository of digital health tools recommended by the organization. The digital tools featured range from mobile apps that time teeth-brushing with music to recordings that coach users through panic attacks. Available online and accessible outside the UK, the NHS App Library models an organized effort to influence the selection and use of mobile health applications. However, it does not regulate development or enforce data security standards. The NHS effort sparked criticism after its first version launched in 2013. In a study that examined privacy risks, reviewers found that 20% of the mobile health apps featured on the NHS Health Apps Library did not have a privacy policy posted, and 78% of information-transmitting applications with privacy policies did not specify what data was shared [14]. The NHS responded by shutting down the library in 2015 before relaunching it in 2017 [15]. The relaunch featured only one NHS-accredited mobile health app and two still in testing [16].

### Revised regulation approaches

These current efforts by both the FDA and NHS represent practical approaches to regulating medical apps and also highlight the challenges of adapting to the fast changing landscape of digital health. Each organization is currently piloting novel approaches that iterate on initial program designs. Today, the NHS Apps Library evaluates resources using a three-step process and a set of Digital Assessment Questions (DAQ) and features a total of 76 applications that address health issues [17]. To add to its volume of accredited digital tools at a faster rate, the NHS is introducing an end-to-end evaluation software that automatically tests for inclusion criteria [18]. An accelerated and less cumbersome approval process makes accreditation more appealing to developers and incentivizes them to design applications that respect basic data privacy rights to begin with. The library is limited, however, to offering advice. In a disclaimer posted on the Apps Library, the NHS excuses itself from any liability and reminds visitors to the webpage that developers are ultimately responsible for the efficacy and safety of the applications they build. The NHS recently collaborated with the National Institute for Health and Care Excellence (NICE) to establish credentials for digital health tools or “Digital Health Technologies” (DHT) [19]. The NICE Framework focuses on the degree to which a DHT is backed by evidence as well as its financial footprint. These standards encourage developers to test software and to build medical technologies with their economic impact in mind.

The FDA similarly revised its approach to medical app regulation to hinge heavily on the role and credibility of the developer. As part of the Digital Health and Innovation Plan, the FDA introduced a “Pre-Certification” program in 2017 for pilot in 2019 [20]. The program vets or “pre-certifies” digital health developers who have already shown credibility and excellence in software design. Applications built by pre-certified developers are exempt from the standard testing and accreditation review. If a developer is given Pre-Cert status, its output is FDA approved. The Pre-Cert program accelerates production, and the benefits that digital health software promises—to patients, doctors, developers, and corporations—materialize at a faster rate.

While these revised approaches are still developing, they have already been met with challenges. Several US senators outlined their concerns with pre-certification in a 12-page letter addressed to the former FDA commissioner and the director of the Center for Devices and Radiological Health [21]. Among them are the criteria that determine a developer’s “excellence,” whether products undergo re-evaluation after they are in use, and who is responsible for maintaining and enforcing regulatory policies across the FDA. These questions target the motivation behind the Pre-Cert program and allege bias towards the digital health marketplace. In an effort to move innovation forward, the FDA has piloted a program that accredits developers and software companies—not the technology itself.

### Efforts to evaluate and educate

As regulatory bodies work towards new solutions, other initiatives have expanded. Independent ratings, decided and published by a range of reviewers, have emerged to measure the value and safety of mobile health and wellness apps. These assessments are widely available and, not unlike the digital tools they evaluate, often published without further review or commentary.

A recent review paper examined several mental health app evaluation websites including Psyberguide, MindTools.io, and ORCHA [22]. The paper highlights a lack of concordance between ratings of the same apps across the various evaluation websites. This is explained in part as these review websites struggle to keep pace with the rapid turnover and rate at which apps are updated and new versions are released. The average age of a Psyberguide review was reported to be 598 days—well over a year old [22]. These scores offer even less value as they are calculated on measures such as “subjective quality” and “perceived impact.” Thus, questionable validity and reliability of scoring criteria, combined with infrequent updates to reviews, renders these recommendations likely inaccurate as well as out of date [23].

A different approach is to help people make more informed decisions about selecting apps without endorsing or recommending a particular one. This approach, developed in part with the authors of this paper, is reflected in the American Psychiatric Association (APA) app evaluation framework [24]. Recognizing that apps are tools and their use will vary by the patient at hand, their clinical needs, and the treatment plan, the framework offers a scaffold for finding and selecting an app that is useful and safe. It suggests that users ask questions across four areas, in order of descending importance: safety and privacy, evidence, ease of use, and interoperability. In learning and determining answers to questions on topics ranging from supporting evidence and claims to the use of personal information, patients and clinicians reach conclusions that meet their individual needs. Equipping patients, clinicians, and the wider public with a way to evaluate digital tools, however, does not placate the need for app regulation. Education and evaluation should not replace regulation and ongoing efforts by the FDA or NHS, but instead supplement them.

### A self-certification design

The previously mentioned models that monitor and regulate medical apps are well intentioned. Their differing approaches and perspectives stimulate conversation among diverse stakeholders and encourage debate on future policies. Today, however, it remains difficult to confidently select a safe and effective mental health app. Efforts and programs from the NHS and FDA will continue to evolve and improve with time, but there is an imminent need to assist clinicians and patients in the meantime. The APA app evaluation framework customized to local needs offers potential, although its use requires that app data presented in the marketplaces and scientific literature is accurate, easily accessible, and up-to-date. This is unfortunately not always the case, with recent studies demonstrating that many mental health apps do not disclose accurate information on how they handle, secure, or store patient data [8]. This lack of transparency, complicated by unmeasured and unfounded claims of many apps [25], makes evaluation cumbersome and time consuming. Likewise, the high rate and frequency of which apps undergo updates necessitates regular reconsideration and rereview. Patients and clinicians need a resource that offers valid and recent information.

**Fig. 1:**
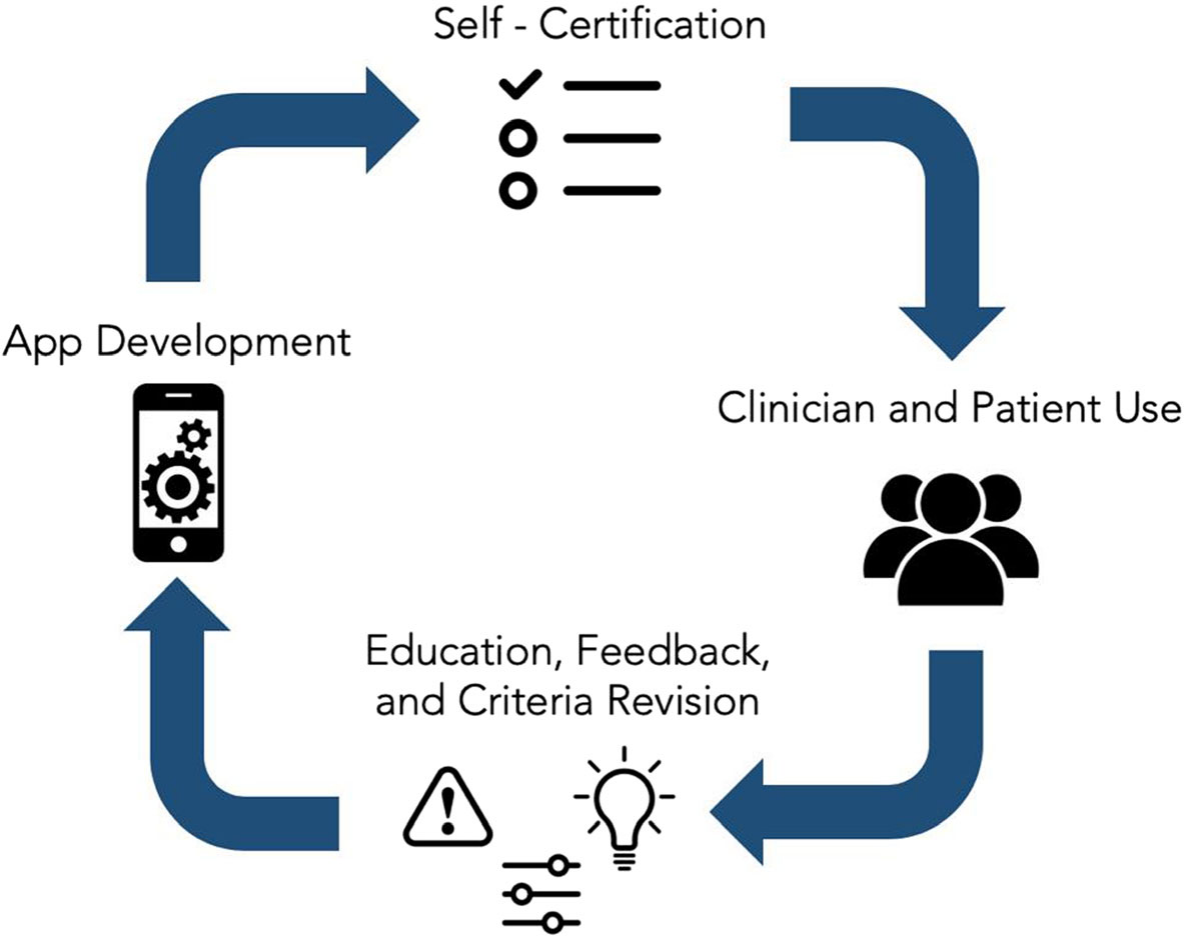
A schematic of the self-certification system towards improving transparency and empowering patients, clinicians, and technology developers to take an active role in regulating digital health tools

As a practical solution aimed towards offering patients and clinicians today useful information about medical apps, we propose supplementing the APA evaluation framework with a self-certification checklist (see Fig. 1). Drawing from the NHS App Library’s approach in assessing inclusion criteria, developers would answer a set of questions about their app—in this case derived from the APA evaluation framework and adapted to contextual needs with diverse stakeholder input. The questions would not be exhaustive but rather focused on practical information patients and clinicians need to know to select suitable apps. Developers’ answers to the self-certification checklist would be publicly available, giving users an opportunity to comment on validity of answers or propose changes to scoring, drawing on the real-world evidence approach that is central to the FDA’s Pre-Cert program. This public, interactive approach to collecting data would hold developers accountable, generate discussion, and create transparency. For example, a patient could filter categories for app choices that meet their standard of privacy, offer a certain level of evidence, are usable based on peer reviews, and present the necessary degree of clinical integration. As with the APA evaluation framework, the goal is not to offer a “top” or “best app” but rather a range of options justified by up-to-date and transparent data.

A self-certification program would also offer ongoing education and teaching. Both patients and clinicians would learn which app features are most appealing, how to flag concerning apps for additional review, and what ways other users are utilizing apps to improve health. Because the FDA and NHS efforts, and logic, dictate that it is impossible to evaluate each mobile app, random audits as well as audits triggered by patient and clinician concerns would be conducted. Any app that completes self-certification is subject to review by the FDA without warning. Given the rapid pace of app evaluation, self-certification would require renewal every 3 months. For such a system to be most effective, it would require app developer buy-in. In a model similar to how Google now enforces certain standards for advertisements posted by rehabilitation and substance abuse facilities [26], self-certification would be a pre-requisite for inclusion in Google Play or the App Store’s library. In the event that a developer has misrepresented information and an app fails an audit, Google Play or the App Store would suspend the app from its library for 3 months until it completes a second self-certification that is determined valid by the FDA. The purpose of this self-certification checklist is not to compete with the FDA approach, but rather augment it with support from leading technology companies and insight from patients and clinicians. The main steps in this self-certification plan as well as advantages and challengs are shown below in Table 1. The assembled volume and range of feedback on the medical apps and on the self-certification process itself would offer useful data to help inform a final version of the FDA’s pre-certification program.

**Table 1:** A table outlining the self-certification process and the significance of key events

Self-certification step	Advantages	Challenges
I. Developers complete self-certification checklist	Motivates app developers to build secure and effective apps that pass the checklist	Developers can misrepresent an app and/or its capabilities and privacy policies
II. App libraries offer self-certified apps publicly available for download	Engages private sector and incentivizes developers to self-certify for inclusion in major app libraries	May slow the rate at which new apps and updates are recommended
III. Apps are subject to community ratings and random audits on accuracy of self-certification report	Facilitates cross-sector and multi-stakeholder collaboration	Ratings are public, giving voice to potentially inaccurate or harmful user content
IV. Developers renew self-certification every 3 months	Ensures app updates and new versions are in line with self-certification policies	Frequency at which apps can be audited requires more reviewers and effort

## Conclusion

The measure of success for any approach to medical app regulation is patient safety. A self-certification program engages policy makers, developers, and patients and clinicians in a learning system that transparently offers as much information as it collects. Such a mutually beneficial interchange prompts the design and build of mobile health apps that meet and respond to real needs. Self-certification sets a standard for transparency that holds developers accountable and incentivizes them to provide accurate information and protect user data. Self-certification also empowers patients and clinicians to play an active role in shaping the future of digital health and ensuring their needs guide the next generation of safe, effective, engaging, and clinically impactful apps.

## Data Availability

NA
